# Testing Foundations of Biological Scaling Theory Using Automated Measurements of Vascular Networks

**DOI:** 10.1371/journal.pcbi.1004455

**Published:** 2015-08-28

**Authors:** Mitchell G Newberry, Daniel B Ennis, Van M Savage

**Affiliations:** 1 Department of Biomathematics, David Geffen School of Medicine, University of California Los Angeles, Los Angeles, California, United States of America; 2 Department of Radiological Sciences, Biomedical Physics, and Bioengineering, University of California, Los Angeles, Los Angeles, California, United States of America; 3 Department of Ecology and Evolutionary Biology, University of California, Los Angeles, Los Angeles, California, United States of America; 4 Santa Fe Institute, Santa Fe, New Mexico, United States of America; Georgia Tech, UNITED STATES

## Abstract

Scientists have long sought to understand how vascular networks supply blood and oxygen to cells throughout the body. Recent work focuses on principles that constrain how vessel size changes through branching generations from the aorta to capillaries and uses scaling exponents to quantify these changes. Prominent scaling theories predict that combinations of these exponents explain how metabolic, growth, and other biological rates vary with body size. Nevertheless, direct measurements of individual vessel segments have been limited because existing techniques for measuring vasculature are invasive, time consuming, and technically difficult. We developed software that extracts the length, radius, and connectivity of *in vivo* vessels from contrast-enhanced 3D Magnetic Resonance Angiography. Using data from 20 human subjects, we calculated scaling exponents by four methods—two derived from local properties of branching junctions and two from whole-network properties. Although these methods are often used interchangeably in the literature, we do not find general agreement between these methods, particularly for vessel lengths. Measurements for length of vessels also diverge from theoretical values, but those for radius show stronger agreement. Our results demonstrate that vascular network models cannot ignore certain complexities of real vascular systems and indicate the need to discover new principles regarding vessel lengths.

## Author Summary

Vascular networks distribute resources and constrain metabolic rate. Founded on a few key principles, biological scaling theories predict characteristic patterns for vascular networks as they branch from large to small vessels. These theories also predict seemingly unrelated phenomena, such as size limits on mammals. However, vascular networks are difficult to measure because there are billions of vessels that range in size from meters to micrometers. To test the foundations of biological scaling theories, we developed software that quickly measures thousands of *in vivo* vessels based on MRI. Data for vessel radii match predicted patterns but lengths do not. Our work suggests the need for new theoretical principles and should facilitate comparisons across organisms, spatial scales, and healthy and diseased tissue.

## Introduction

Networks that supply resources are essential to the maintenance and growth of many natural and engineered systems. These resource-distribution networks are pervasive throughout biology. Examples include the tracheal system in insects, xylem networks in plants [1], foraging trails of ant colonies [2, 3], and cardiovascular systems in animals [4–6]. Because the cardiovascular system delivers resources and energy to the body, its structure at least partly determines rates of growth and metabolism [4, 7, 8]. Moreover, the cardiovascular system plays a role in many diseases—such as heart disease, stroke, inflammation, and malignant tumor growth [9–11]. The pervasiveness and importance of material transport in biology motivates the search for basic principles that help shape resource-distribution networks in general and the cardiovascular system in particular.

The theory of the structure of vascular networks has roots in the early 20th century [5, 7, 12]. More recent theories predict properties of the entire vascular network by assuming a hierarchical structure and include those of West, Brown and Enquist [4] (henceforth the WBE model), Banavar et al. [13, 14], Dodds [15], Huo and Kassab [16], and others [1, 17–21]. These theories assume or predict how vascular structure, such as the radius of vessels, changes as the network branches from aorta to capillaries. While early theories focused on vessel radius, recent theories also incorporate how vessel length changes across the network [4, 14–16]. Many also assume symmetric branching—child vessels have identical properties [4, 16, 19, 20]. In symmetric, hierarchical models, knowledge of both radius and length enables derivations of how metabolic rate varies with body size across species [4] and how organismal and tumor growth rate change with body size [9–11].

Several theories predict vascular structures will be found to be self-similar—some aspect of the network can be viewed as a rescaled copy of the whole [22]—across specific ranges of spatial scale [4, 23]. Self similarity necessarily leads to relationships and distributions that are characterized by power laws, whose exponents we call the scaling exponents [24]. Self similarity can either be a strict or statistical property. Each new chamber of the nautilus is a larger exact copy of the previous chamber, while along coastlines shorter segments exhibit rescaled statistical properties of the longer segments [25]. Self similarity in nature suggests scale-free principles that constrain structure. An organism may need to maintain a certain shape at all stages of growth, use a common developmental program at all scales, or cope with physical processes with no preferred scale—such as energy minimization or turbulence in fluids. Self similarity greatly simplifies many calculations, with applications from cardiac physiology [26] to allometric scaling [4].

Real vasculature is known to deviate from hierarchical, symmetric models in many ways, leading to criticism and debate about leading models [27, 28]. Without reliable data, it is impossible to determine whether these deviations can be ignored, so that existing theories can accurately predict newly observed features such as curvature in scaling relationships [19]. Price et al. [29] recently decried the lack of data for individual vessel segments that are needed for tests of scaling theory. Direct measurements and tests may reject existing principles while laying the groundwork for the discovery of new patterns and principles in vascular architecture. In addition, measurements can parameterize existing equations to obtain more exact predictions for metabolic rates and growth curves, and can be used to examine the natural variation in the parameters across species, tissues, and tumor types. Vessel segment data preserves the asymmetry, reticulation, tortuosity, and other features of real vasculature, and quantitative data about these features may inform future models. Furthermore, extreme values or distinct patterns of variation may be signatures of pathologies that could eventually be used as diagnostics. Recent work involving direct measurements of plant architecture has begun to realize this potential [21, 30, 31].

Comprehensively characterizing vascular structure and obtaining reliable estimates for vascular scaling exponents requires large numbers of measurements across orders-of-magnitude in spatial scale—ranging from 0.004 mm to 15 mm for vessel radii in humans. Measurement is complicated because vascular systems are intertwined with tissues throughout the body at a wide range of spatial scales [32]. Even gross morphological measurements have historically taken impressive and time-consuming efforts and required invasive methods such as casting [33–36]. Zamir [36] and Kassab [35] constructed explicit descriptions of small regions of vascular systems—such as the coronary artery—by perfusing fixed specimens with a silicone or acrylic polymer, dissolving tissue away and examining each vessel segment under a microscope. These approaches enabled the measurement of tens of thousands of vessels from fixed specimens that were then used to test and develop vascular system models [35, 36].

However, the process of casting may enlarge or damage vessels, and little of this raw data is publicly available for analysis. In addition, many more measurements across the range of scales are needed to identify the principles that shape vascular architecture. Different physical principles may dominate at different scales, and mapping out different regimes will require large amounts of data at each scale. In the WBE model [4], the dominant mechanism of energy loss for blood flow in the arterioles (radius ≪ 1 mm) is viscous dissipation, but near the heart (vessel radius ≫ 1 mm) pulsatile flow and reflection of pressure waves along vessel walls dominates [26]. The transitions between these regimes are neither well-understood theoretically nor described empirically [8]. The pulsatile regime—the focus of our measurements—has greater variation in the number of branching orders and size of vessels across species and is thus the primary determinant of scaling of metabolic and other vital rates with body size [8].

Large amounts of vascular data across all relevant spatial scales are contained within existing angiographic images (e.g., MRI and X-Ray). These images are obtained non-invasively, thus avoiding problems of damage to vasculature and allowing for the possibility of longitudinal studies. The latent data within these images represents a tremendous opportunity. All that is needed is a reliable and automatic method for extracting vascular data from angiographic images.

We have developed novel software that imports 3D images, creates a topologically and spatially explicit map of the blood vessel network, and measures the radius, length, and volume of all visible vessels. We have applied this software to 20 magnetic resonance angiograms of living humans to obtain 3015 data points that range in radius from 0.6–6.8 mm, representing an order of magnitude. This range corresponds to large vessels in the pulsatile flow regime relevant to allometric scaling [8]. We then analyzed these data based on the four distinct methods (Eqs 1a–4) for measuring vascular scaling exponents described below.

## Model

Vascular scaling exponents encapsulate how radius and length of vessels change across the network. Virtually all scaling relationships for local or global properties can be expressed in terms of these vascular scaling exponents. Consequently, we view these scaling exponents as forming the foundation of most modern biological scaling theory and make them the primary focus of our analysis. We here describe four distinct methods for calculating vascular scaling exponents.

Because the radius of the vessel plays a primary role in determining both the flow rate and resistance to blood flow through the vessel, theories for the vascular scaling exponent for vessel radius often focus on the power to pump blood from the heart to the capillaries. It is argued [4] that this power will have been minimized by natural selection to allow as much power as possible to be available for foraging, growth, and reproduction. One classical approach minimizes power loss of blood flow due to viscous dissipation and due to cost of blood volume in order to derive that flow rate, $\dot{Q}$, depends on the cube of the vessel radius, *r* ^3^. Combining this result with conservation of fluid flow at a branching junction yields Murray’s law, $r_{p}^{3}=\sum_{i}r_{c,i}^{3}$, where *r*_*p*_ is the radius of the parent vessel segment and *r*_*c*,*i*_ is the radius of the *i*th child (distal) segment. Another classical approach for the cardiovascular system is to minimize wave reflections in pulsatile flow, as Womersley and West et al. have done [4, 6, 26]. This approach leads to area-preserving branching (or Da Vinci’s Rule), so that the sum of the cross-sectional areas (∝ *r* ^2^) of child vessels equals the cross-sectional area of a parent vessel at a branching junction. In the WBE model, reflections dominate for large vessels while dissipation dominates for small vessels (≪ 1 mm). Moreover, in the WBE model, a volume-servicing argument [4] is used to derive an analogous relationship for vessel lengths, $l_{p}^{3}=\sum_{i}l_{c,i}^{3}$, while Huo and Kassab assume the same relationship but allow the exponent to vary from length-preserving (exponent of 1) to volume-servicing (exponent of 3).

Optimization has been a common approach to developing vascular models throughout the past century, but it has been highly debated as to which properties are optimized and what are the tradeoffs among them [4, 27, 28, 37–39]. For instance, Banavar et al. [40] optimize for efficient transport within three-dimensional bodies and Dodds [15] minimizes network volume as required to continually supply metabolites within a body. Indeed, different principles and assumptions lead to a variety of relationships between the flow rate and vessel radius. Consequently, we express a generalized form of Murray’s law

$$r_{p}^{1/a}=\sum_{i}\;r_{c,i}^{1/a}.$$ (1a)

in which we define the vascular scaling exponent, *a*, for vessel radius to be consistent with the notation of Price et al. [1]. The analogous generalization for vessel length is

$$l_{p}^{1/b}=\sum_{i}\;l_{c,i}^{1/b}$$ (1b)

where *b* is the vascular scaling exponent for vessel length.

To ease computation, many models further assume that vascular networks are strictly self-similar and symmetrically branching—child vessels all have identical properties within a branching level. In this case, scale factors and associated scaling exponents can be defined for each branching level, *k*, which represents the number of branching junctions from the heart to that vessel. Following the notation of the WBE model, the scale factors are *β* = *r*_*k*+1_/*r*_*k*_ and *γ* = *l*_*k*+1_/*l*_*k*_. For dichotomous branching, we can solve for *β* and *γ* using Eqs 1a and 1b, to find

$$\beta =\frac{r_{k+1}}{r_{k}}=2^{-a}\;\text{and}\;\gamma =\frac{l_{k+1}}{l_{k}}=2^{-b}.$$ (2)

Furthermore, for these idealized networks, the frequency distributions of radius and length follow power laws with scaling exponents 1/*a* and 1/*b*. Because there are *N* = 2^*k*^ vessels of radius *r* = *r*_0_ *β* ^*k*^, Eq 2 give the two power-law relationships

$$N={\left(r/r_{0}\right)}^{-1/a}={\left(l/l_{0}\right)}^{-1/b}.$$ (3)

Similarly, for any vessel, its radius and length are related to the number of downstream endpoints (e.g. capillaries), *N*_*d*_, by

$$r\propto N_{d}^{a}\;\text{and}\;l\propto N_{d}^{b}.$$ (4)

Eqs 1a–4 constitute four methods of calculating vascular scaling exponents. Each method relies on different types and levels of information. First, for each branching junction, the generalizations of Murray’s law (Eq 1a) and volume servicing (Eq 1b) can be numerically solved for the exponents *a* and *b* by Newton’s method. The solution provides a direct local measurement of the scaling exponents at every junction that we call *conservation-based* scaling exponents. The value is undefined if a child vessel has radius or length greater than its parent. Second, for each parent-child pair of vessel segments, the ratio of vessel radius and length can be calculated. By Eq 2, these scale factors can be used to compute a second local measure—our *ratio-based* scaling exponents. Third, across all vessels and junctions, empirical distributions of radii and lengths can be fitted to power laws to produce what we term the *distribution-based* scaling exponents, as in Eq 3. Fourth, across all vessel segments, log-log regressions of radii and lengths versus the number of downstream endpoints can be performed to derive *regression-based* scaling exponents, following Eq 4. These latter two methods each provide single values for the vascular scaling exponents, *a* and *b*, across the whole network, and they do not rely on information about the geometry of individual branching junctions.

In the literature, these four methods for measuring scaling exponents are often used interchangeably [1, 29, 41, 42]. However, these are only proven to be identical for symmetrically-branching, strictly self-similar networks. Furthermore, it is unknown whether measurements of vascular scaling exponents using these four methods (Eqs 1a–4) will produce values that are approximately similar or significantly different. If they differ, this raises questions about which of these four measures of vascular scaling exponents, if any, best corresponds to the scaling relationships predicted by ideal networks or observed empirically for metabolic and growth rates.

## Results

To begin to answer these questions, we now report results obtained by applying our new software, angicart, to 20 contrast-enhanced 3D Magnetic Resonance Image volumes for human head and torso. We collected 3015 segments across 1473 branching junctions. Of the junctions, 1422 were recorded as dichotomous and 51 as trichotomous. The number of branches on all paths (subjects pooled) between the aorta and the smallest observable vessels was typically between 3 and 10 (middle 68%). Each 3D image volume, corresponding to 151 segments on average, took about 2.7 minutes of CPU time on a single 2.3GHz Intel processor. As described in the Methods, we used an automated circularity criterion as an indicator of possible errors in order to omit vessel identification errors. Our results are based on radius, length, and volume measurements from only the 1240 segments for which we saw no indication of potential error.

The complete, raw output of our software before any filtering or analysis is available as S1 Dataset. The software source code and original imagery enabling the exact reproduction of this dataset are available online as a repository in the git revision control system at https://github.com/mnewberry/angicart.

### Conservation-Based Exponents

We attempt to solve Eq 1a numerically at each branching junction. We label the parent at each branching junction by traversing the vascular network starting at the vessel segment of greatest radius, which we assume is a segment in the aorta. If all children are smaller than the parent, the equation is guaranteed to have exactly one solution, and numerical convergence to the solution is fast. Otherwise, the equation may have zero, one, or two solutions. Such cases may represent real anatomical variation, or misidentification of the parent vessel due to errors or ambiguous topology such as the Circle of Willis. We consider only the simplest case, in which children are smaller than their parent, because otherwise the solutions are difficult to interpret in the context of existing vascular network models. All child radii are smaller than the parent in 82% (222) of junctions, and all child lengths are smaller in only 35% (94) of junctions.

**Fig 1:**
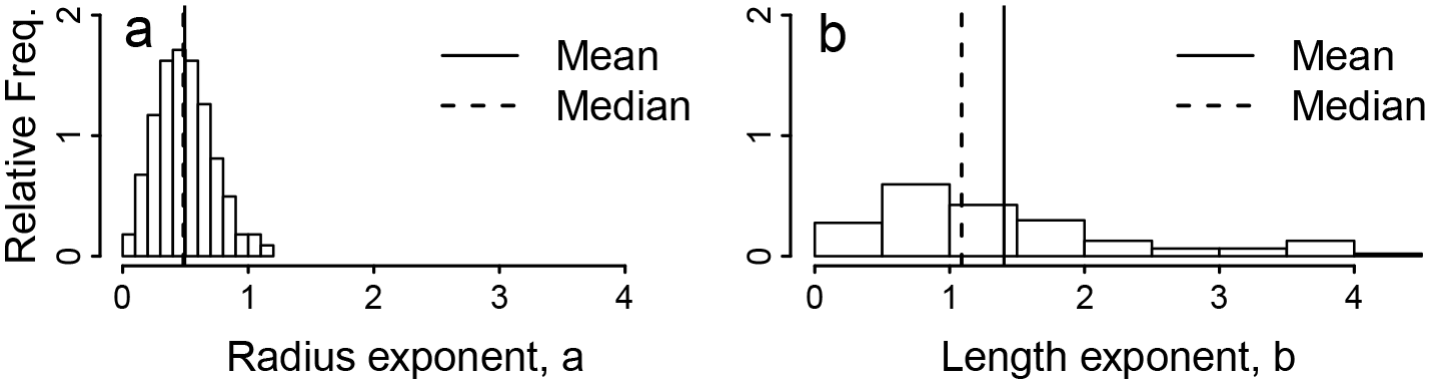
Conservation-Based Exponent Distribution. Plots of (a) the frequency distribution of the vascular scaling exponent *a* for vessel radius from solutions to Eq (1a) using empirical measurements of vessel radii extracted from magnetic resonance angiography using our software, and (b) the analogous frequency distribution of vascular scaling exponent *b* for vessel segment length.

We show distributions of these measures of the vascular scaling exponents, *a* and *b*, across branching junctions in Fig 1. The arithmetic mean and associated 95% confidence intervals for these conservation-based measurements of *a* and *b* are presented in Table 1.

**Table 1:** Measures and theoretical values of the scaling exponents *a* and *b*. *N* denotes the number of measurements incorporated in the average or slope. The 95% CI indicates the range of confidence on the average or slope. For node-level measures, *σ* indicates the standard deviation of measurements made for individual junctions or parent-child pairs for conservation- and ratio-based measures respectively. For network-level (regression- and distribution-based) measures, *R* ^2^ denotes the correlation in the fit. For distribution-based measures, this correlation is between the natural log of bin size and mean vessel dimension (i.e., radius or length) in the bin. For regression-based measures, this correlation is the correlation between the natural log of dimension and the natural log of the number of downstream endpoints. For conservation-based measures, *N* counts all junctions of three or more well-segmented vessels (see Software and Algorithm) for which Eqs 1a or 1b had a solution. For ratio-based exponents, *N* counts all parent-child pairs of vessels in which both the parent and child are well-segmented. For distribution-based exponents, *N* counts the number of well-segmented vessel segments exceeding the minimum-size threshold described in Data Fitting. For regression-based exponents, *N* counts all well-segmented vessels. The 95% CIs are derived in a manner appropriate to each method: They are 1.96 times the standard error on the mean for conservation- and ratio-based measures and the confidence interval on the SMA regression slope for regression-based measures. For distribution-based measures, they are the range of the middle 95% of slopes derived from alternative binning as described in Data Fitting.

**Radius Exponent Measures**	*N*	*a*	95% CI	*σ*	*R* ^2^
Conservation (Node)	222	0.49	± 0.03	0.23	–
Ratio (Node)	703	0.43	± 0.03	0.39	–
Distribution (Network)	657	0.30	± 0.04	–	0.90
Regression (Network)	1240	0.41	± 0.02	–	0.66
**Theory**					
WBE Small Vessels	–	0.33	–		
WBE Large Vessels	–	0.50	–		
Banavar et al. [14]	–	0.50	–		
Huo and Kassab [16]	–	0.33–0.50	–		
Murray’s Law	–	0.33	–		
**Length Exponent Measures**	*N*	*b*	95% CI	*σ*	*R* ^2^
Conservation (Node)	94	1.40	± 0.20	0.98	–
Ratio (Node)	703	0.17	± 0.12	1.57	–
Distribution (Network)	518	0.73	± 0.10	–	0.68
Regression (Network)	1240	0.94	± 0.05	–	0.19
**Theory**					
WBE All Vessels	–	0.33	–		
Banavar et al. [14]	–	0.50	–		
Huo and Kassab [16]	–	0.33–1.00	–		

### Ratio-Based Exponents

**Fig 2:**
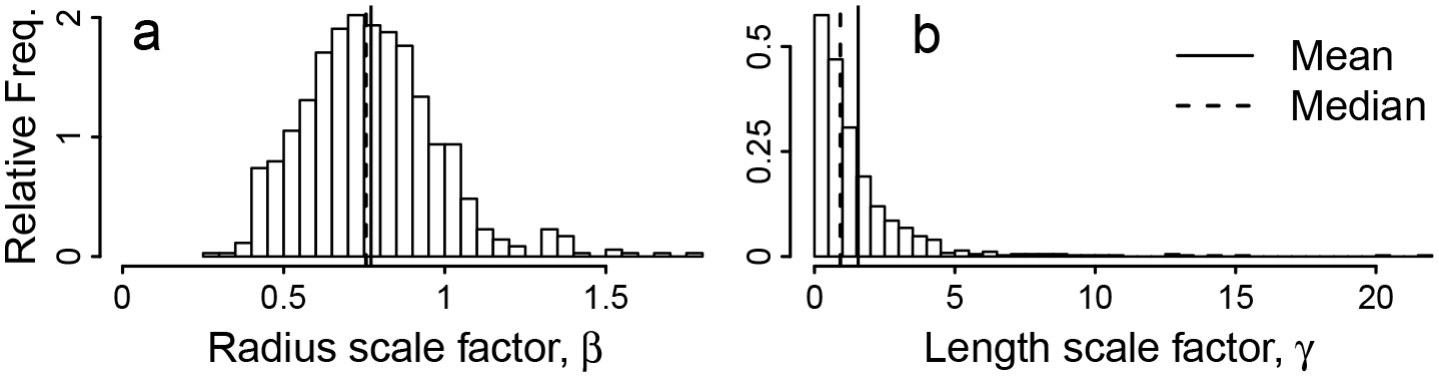
Ratio-Based Exponent Distribution. Plots of (a) frequency distribution of scale factor *β*, the ratio of child to parent radii, and (b) the analogous distribution of scale factor *γ*, the ratio of child to parent lengths.

Topological information allows us to compute *β* and *γ* directly for each parent-child pair of vessel segments. Our dataset contains 703 pairs of parent and child segments with dichotomous branching. A small proportion of the *β* and *γ* values may be over-estimated due to misidentification of the parent-child relationship (see Methods). This bias would produce underestimates in *a* and *b*, but because misidentification is very infrequent, the magnitude of this bias is expected to be within the measurement error. The distribution of *β* and *γ* is displayed in Fig 2, and the arithmetic mean and associated 95% confidence intervals of the ratio-based vascular scaling exponents calculated using Eq (2) are shown in Table 1.

### Distribution-Based Exponents

**Fig 3:**
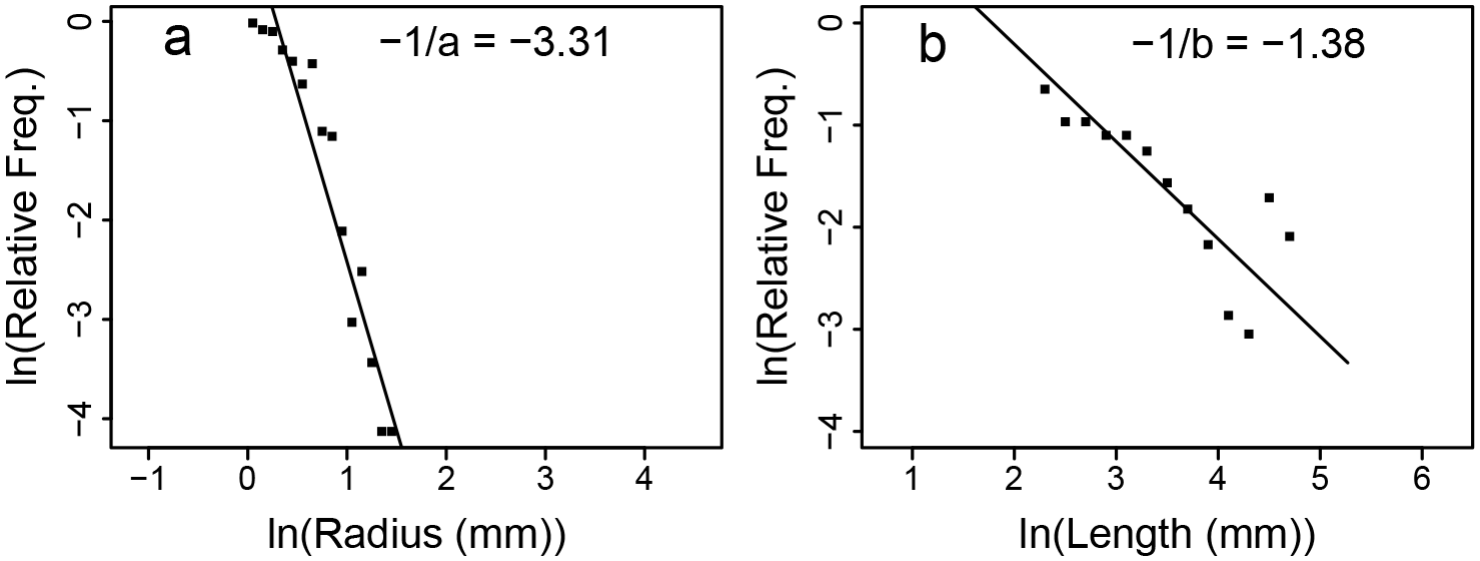
Distribution-Based Exponent Fits. Standard Major Axis regression (see Methods) of the natural log of relative frequency (probability density) against (a) the log of radius, ln *r*, and (b) the log of length, ln *l*. Fit lines and slope values are shown. The correlation coefficient (*R* ^2^) for the log relative frequency versus the logs of radius and length respectively are 0.90 and 0.68.

For symmetrically branching, self-similar networks, the frequency of radius and length measurements follow power-law distributions. We did a linear fit to the log-log transformed histograms of radius and length measurements (the log of Eq (3)) using SMA regression. We derived empirical 95% confidence intervals by resampling with different bin sizes and cutoff values for the tail (see Data Fitting). The fits are shown in Fig 3. The scaling exponents obtained from our fits are given in Table 1.

### Regression-Based Exponents

**Fig 4:**
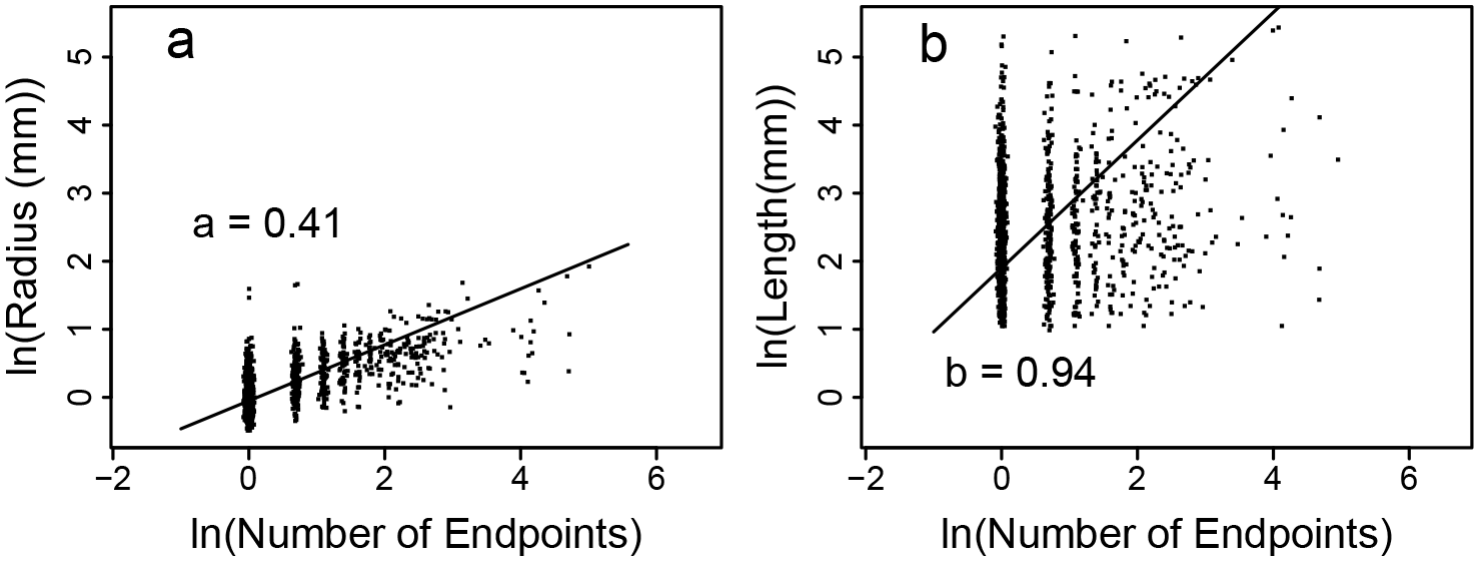
Regression-Based Exponent Fits. Standard Major Axis regression (see Methods) of (a) the log of radius, ln *r*, and (b) the log of length, ln *l*, against the log of the number of downstream tips, ln *N*_*d*_. Fit lines and slope values are shown. Correlation coefficients (*R* ^2^) are 0.66 and 0.19, respectively (*P* < 0.01 for each).

By taking the logarithm of Eq (4), we can estimate the vascular scaling exponents, *a* and *b*, by performing regressions of the logarithm of the number of downstream tips ln *N*_*d*_ against the logarithm of radius, ln *r*, and the logarithm of length, ln *l*, respectively [41]. Regression lines are shown in Fig 4. The measured slopes, which are the estimates of the vascular scaling exponents, and associated 95% CI are shown in Table 1.

### Sensitivity Analysis

As in all analyses based on experimental data, measurement errors affect uncertainties in quantities calculated from the raw data. Consequently, we investigate the sensitivity of our calculated scaling exponents and entire analysis to the choice of threshold intensity in our algorithm as well as to Gaussian noise in the image quality.

**Fig 5:**
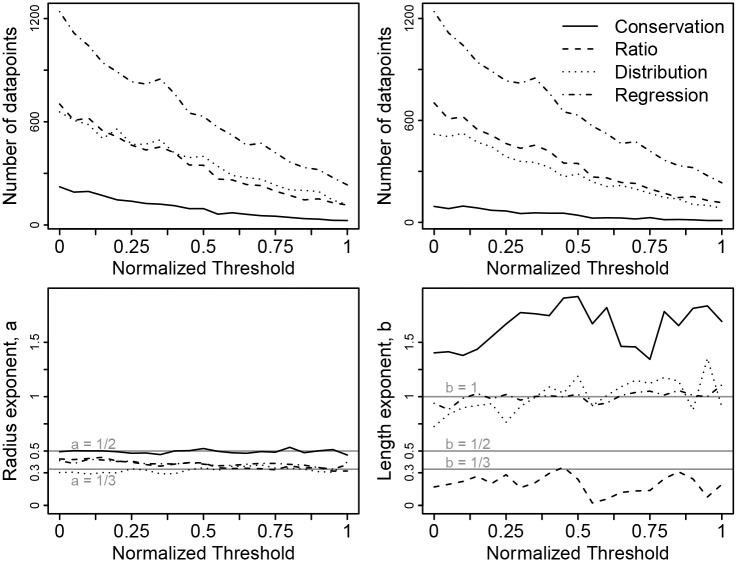
Sensitivity of the measured scaling exponents to the threshold intensity values that select which voxels are part of the vessel lumen. We normalized the threshold so that the minimum threshold (0.0) is the value used in our results and figures above, while the maximum threshold (1.0) is the largest value for which at least 30 vessel segments are visible. We re-scaled the threshold values because the raw value is different for each image. The top left panel shows the number of vessel segments used to calculate the four scaling exponents for vessel radius versus the normalized threshold value, as in the *N* column of Table 1. The top right panel shows the number of vessel segments used to calculate the four scaling exponents for vessel length versus the normalized threshold value, as in the *N* column of Table 1. For both of these top two panels, the number of data points decreases with threshold value as it must. The bottom left panel depicts how the four calculated scaling exponents for vessel radius vary with normalized threshold value. The bottom right panel depicts how the four calculated scaling exponents for vessel length vary with normalized threshold value. Horizontal lines indicate points of comparison to theory as described in Table 1.

An intensity threshold is used in our algorithm to select the voxels from the image that describe the shape of the vessel lumen. Lower thresholds reveal more vessels, so for our analysis above, we used the minimum threshold that produced reliable segments, as described in Software and Algorithm and S1 Text. Because the threshold affects the boundaries of the vessel lumen, the vessel radius also depends on the threshold. For each image there is a minimum acceptable threshold that we used above, and for our sensitivity analysis, we also chose a maximum threshold to be the largest value for which at least 30 vessel segments are visible. For our plots in Fig 5, we normalized the threshold to range from 0 and 1. That is, 0 is the threshold used for the results above, and 1 is the maximum threshold for which at least 30 vessel segments are visible. For each normalized threshold increment of 0.05 between 0.00 and 1.00, we ran our entire analysis and calculated the four scaling exponents for radius and length (Fig 5). The values of the scaling exponents at a normalized threshold of 0.00 recapitulate Table 1. Our results remain qualitatively similar as we increase the threshold and include fewer segments. However, at higher normalized thresholds, we can no longer resolve differences between some exponents that are resolvable at normalized threshold 0.00, as expected from the law of large numbers.

Noise in images may also cause errors in the identification of vessels or in estimates of the radius or length of vessels. We conducted a sensitivity analysis similar to the above by adding Gaussian noise that varied in magnitude from 0.47 (the measured baseline level of noise in the foreground of one image) to 4.7% (10 times the baseline noise level) of the maximum voxel intensity. Results (S1 Fig) show no significant changes in our results with higher levels of noise.

As another measure of uncertainty, we located vessels with radius estimates that differed with threshold despite high reliability in vessel identification (vessel endpoints were similar across at least 5 threshold values). We located 12 vessel segments from 9 different patients that matched this criterion. Across these 12 vessels, the mean radius estimate varied from 0.9 to 6.7mm, but the coefficient of variation (= standard deviation/mean across thresholds) ranged only from 0.02 to 0.08. Moreover, the coefficient of variation was uncorrelated with radius (Pearson correlation = 0.07), suggesting that individual vessel radius measurements are precise to roughly ±10% (2 times the coefficient of variation) regardless of vessel size. For a specific threshold, we calculate how the measured vessel radius differs from the mean of the vessel radius across all thresholds. At each threshold, this difference tended to be in the same direction (mostly positive or mostly negative) across the 12 vessels we measured. Moreover, the magnitude of the difference was proportional to vessel radius (most *R* ^2^ > 0.99). Consequently, the ratio of vessel radii at any specific threshold is roughly equal to the ratio of the means of the vessel radii across thresholds and also equal to the ratio of vessel radii at any other specific threshold. That is, within the plausible range of thresholds that we explore here, the calculated scaling ratios are largely independent of the choice of threshold value, implying that the choice of threshold does not create any bias in the calculated scaling exponents or ratios.

Of course, our sensitivity analysis cannot exclude all possible sources of error or bias. In the Methods sections, we discuss how our results might be affected by other possible sources of error, such as tree topology identification, the patient population, small vessel censoring, vessel lumen misidentification, skeleton line selection, centerline quantization, and vessel segment misattribution.

The positional accuracy of MRI is high, so errors arise due to classification or interpretation of voxels. Because the threshold parameter and noise in the image primarily control how we classify voxels—the first step of analysis—these are the major determinants of subsequent errors. In our analysis, as presented in Fig 5 and S1 Fig, no systematic biases are observable, so we conclude that our results are highly robust to the largest and most notable sources of uncertainty.

### Comparing Measurements with Each Other and Predictions

The estimated values of vascular scaling exponents obtained using our four different methods are all presented in Table 1. All pairs of measures are statistically significantly different (Welch’s t-test, *P* < 0.01) except for the ratio-based and regression-based *a* (*P* = 0.18). Values of *a* based on conservation rules at branching junctions, scale factors for parent-child pairs, and regression of ln *r* versus ln *N* are all between *a* = 1/2 (the WBE prediction for large vessels) and *a* = 1/3 (Murray’s law, the Banavar et al. prediction [14], and the WBE prediction for small vessels), and the remaining distribution-based *a* includes *a* = 1/3 in its 95% CI. The conservation-based exponent *a* is not statistically-significantly different from *a* = 1/2.

Different measures for *b* range from 0.17 to 1.40 and all are statistically significantly different (*P* < 0.01) from each other and from the volume-servicing and area-servicing values of *b* = 1/3 and 1/2 respectively. It is notable, however, that the difference between regression-based and distribution-based measurements of *b* is no longer resolvable at normalized thresholds higher than 0. The distribution-based and regression-based exponents lie between area-servicing and length-preserving. These discrepancies between measures suggest that vessel segment lengths are poorly modeled by strictly self-similar and symmetrically branching networks.

## Discussion

Our software acquired direct measurements of a large number of connected vessel segments from *in vivo* angiography. We calculated vascular scaling exponents in these data using four methods to directly compare values from real vascular networks with each other and with theoretical values from the WBE model, Murray’s Law, Banavar et al. [14], and Huo and Kassab [16]. Intriguingly, our results lead to contrasting conclusions for the changes in vessel radius and length across scale.

For the vascular scaling exponent *a* that quantifies changes in the radius, the conservation-based and ratio-based estimates are closer to *a* = 1/2 than *a* = 1/3. These estimates support work on large vessels by West et al., Zamir and Banavar [4, 14, 43], in contrast to Murray’s law, which does not distinguish large and small vessels. West et al. derive that the dominant source of power loss for large vessels (estimated to be *r* ≫ 1 mm) is the reflection of pressure waves at branching junctions, while for small vessels, power loss is dominated by viscous dissipation between blood and the vessel walls. Because of the resolution of our MRI volumes, we are able to extract data mostly for vessels with a radius greater than 1 mm, corresponding to the large vessel regime in the WBE model. Consequently, our results for the scaling of radii are supportive of the area-preserving branching of large vessels, corroborate recent findings for plants [41], and reject the possibility that Murray’s law might apply, either generally or on average, to junctions of large vessels. This result demonstrates that minimizing energy-dissipation due to blood flow does not capture the guiding principles that shape the vascular system across all scales. Future studies using higher resolution angiography (e.g., micro-CT) to obtain data for small vessels are needed to test Murray’s law and the WBE prediction for small vessels and to determine if minimizing energy dissipation is a relevant principle at any scale.

For the vascular scaling exponent, *b*, for vessel lengths, the discrepancies between predicted and estimated values are more difficult to reconcile and interpret. None of the four measures of *b* agree with each other or provide support for volume- or area-servicing branching, while only the regression-based method provides support for length preservation. Within the WBE model, *b* is predicted to be 1/3 based on an argument that the vascular network must be volume-servicing for the entire body [4]. This volume-servicing argument has been questioned on theoretical grounds [28], and here we provide empirical evidence that volume-servicing or any other conservation law for length does not hold locally at branching junctions. Indeed, for the conservation-based exponents, 65% of branching junctions violate the model so severely that exponent values are undefined. The volume-servicing argument is supposed to apply across at least the vast majority of scales and is a key element of the WBE explanation for the 3/4 allometric scaling relationship between metabolic rate and body size. The breakdown between this argument and the real vascular networks we measured may occur because, contrary to the WBE argument, the length of a vessel segment is not a reliable indicator of the volume it services. The correlations between length and number of downstream endpoints, a proxy for volume serviced, is very low compared to the same correlation for radius (0.2 versus 0.7). Considering only the largest vessels, this is not surprising. The ascending aorta is only a few centimeters in length and services most of the body, while the carotid artery is much longer (at least 10 cm in length) and services only half the head. Our results imply that either modification of the volume-servicing argument is needed or some new principle yet to be discovered guides the distribution of vessel lengths as the vascular network branches throughout the body. These new developments could lead to corrections to the power-law predictions of the original WBE theory that may agree better with recent findings of “curvature” in the allometric relationship [19].

Beyond the differences discussed thus far, vessel lengths and radii also differ in their distributions for vascular scaling exponents and scale factors. Measurements of *a* and *β* exhibit a strong central tendency (Figs 1 and 2), while the scale factor for length, *γ*, has a highly skewed distribution with typical values that are not well-described by the mean. Thus, a derivation implicitly based on a mean-value approximation may be successful for predicting vessel radii but fail to predict scaling relationships involving vessel lengths. Thus, while hierarchical symmetric models may fail outright to adequately describe vessel lengths, the discrepancies between vessel radii in real networks and idealized models, such as the WBE model, may only result in minor corrections to model predictions. This may help explain the success of the WBE model in predicting a wide range of phenomena.

Differences in results for vessel radius and length could be tied to different strengths of the constraints on vessel geometry. Radii and length distributions have previously been observed to differ in the external branching of plants and leaves [41, 44]. One explanation for this is that viscous power loss depends much more sensitively on vessel radius (as a 4th power, ∝ *r* ^4^) than on vessel length (linearly, ∝ *l*). Thus, the strength of selection for optimal vessel radius is much stronger than for optimal vessel length, implying evolution has more often sacrificed vessel length when negotiating tradeoffs in anatomy. Another potential explanation is that vessel radii are self-similar due to a local constraint at each branching junction, whereas vessel lengths may be constrained only at larger scales—organs and organisms—that more accurately capture how the vascular network needs to span and feed a spatially inhomogeneous body.

Disagreement about the value of the length scaling exponent between our four methods indicates that assumptions of the simplest model must be violated so strongly as not to hold even approximately. That is, strict self similarity, symmetric branching or both must be strongly violated for the real vascular networks we measured. Our data reveal pervasive asymmetry in both radius and length between child vessels. How far the results for symmetric networks generalize to asymmetric networks has been explored very little [45]. The differences we observe between different measures of vascular scaling exponents could be explained by the inability of existing theories to account for the asymmetry of real vascular networks. Developing a theory to account for asymmetric branching may be challenging. For instance, accounting for asymmetry would require at least two scale factors for radii (e.g., *β*_*big*_ and *β*_*small*_) and two for lengths (e.g., *γ*_*big*_ and *γ*_*small*_). These additional scale factors and associated scaling exponents would necessarily change our analysis and our estimates for the ratio-based scaling exponent, and would potentially change our interpretation of the distributions of *β* and *γ*. Rather than thinking of distributions of *β* and *γ*, we would think of joint distributions of *β*_*big*_ and *β*_*small*_, for example. For similar reasons, our interpretation and analysis of the frequency distribution of radius and length could be altered, thus affecting the estimates of the distribution-based exponents as well. Angicart outputs *β* and *γ* values for each vessel pair, and can provide the detailed information required for future studies of multiple scale factors for length or radius and asymmetric branching.

All of the models discussed in this paper ignore any reticulation or loops in the vessel topology, in contrast to recent work on leaf venation networks [46–49]. Our analysis also follows this assumption. However, loops are known to occur anatomically in healthy (Circle of Willis) and diseased (tumors, arteriovenous malformations) tissue. Extensions to our software and to theory could address this issue. Such an extension could be used to investigate abnormal tumor vasculature [9], or allow new theory to be developed to explain the normal anatomical function of reticulation. There are also other spatial aspects that have received theoretical attention, such as branching angle, that our software is already capable of recording. Many more tests could be performed with data on microvasculature. For instance, Huo and Kassab [16] have published scaling relationships for how crown volume and length change with stem radius. Testing these requires knowledge of the full crown, down to the microvasculature. Similarly, Dodds [15] makes predictions for virtual vessels that coincide with real vessels only at the smallest scales.

We developed new software and applied it to MRI of human head and torso to obtain one of the most detailed datasets for examining branching architecture in vascular networks. In addition, we conducted a comprehensive data analysis that uses both local and global methods to measure scaling exponents. Together, this new software, data, and analysis provides valuable information for answering fundamental questions about vascular system morphology. The public release of our imaging software, angicart, should enable researchers to ground future vascular network theories in empirical data. The software facilitates comparison across spatial scales and between studies by operating uniformly on all tomographic imaging methods. Because imaging can be done non-invasively, our method affords the opportunity to record all spatial information of *in vivo* vasculature through time or across development.

We explain four different methods to estimate vascular scaling exponents from spatially-explicit data. Although researchers use and sometimes interchange these four methods, we found that all four methods can lead to different results, and that for scaling exponents for vessel lengths, these differences can be dramatic. This result is in stark contrast to theoretical calculations for idealized, symmetric networks that predict all methods will give identical values. We advise caution when interpreting different methods and estimates as the same scaling exponent because this could lead to misperceptions and disagreements among studies. For instance, regression-based estimates are the most common across levels of biological organization while distribution-based estimates are used for forests [42], so comparing these estimates to each other must be done with care. The differences we observe call for a new understanding of the relationships between the local geometry of vessels and the global properties of vascular systems. New theory should be developed to accommodate the anatomical variation and asymmetric branching we observe in real networks.

## Methods and Materials

### Ethics Statement

After local institutional review board approval and written informed consent had been obtained, 20 consecutive adult patients with clinically suspected supraaortic arterial occlusive disease were prospectively enrolled to evaluate new MRI methods for the study of carotid atherosclerosis [50].

### Image Acquisition

Although other data was recorded in that study, we use only the images. In that study less than 0.5% of observed vessel segments had notable luminal narrowing, so we conclude patient selection and enrollment did not affect our results. We acquired contrast-enhanced magnetic resonance angiograms (CEMRA) of the upper torso, neck, and head in the 20 human subjects (N = 20) using a 3 Tesla Siemens Trio scanner (Siemens Medical Solutions, Erlangen, Germany). The data acquisition details have been previously described [50]. In brief, the CEMRA images were acquired after an antecubittal vein injection of gadolinium based contrast agent (Gd-DTPA, Magnevist, Bayer Shering Pharma AG, Berlin, Germany). The image volumes have dimensions that are typically close to 380 × 640 × 128 voxels, with each voxel nearly isotropic and between 700 × 700 × 800 μm and 800 × 800 × 900 μm. The resolution and imaged volume are typical of high-quality 3T MRI. The point-spread-function for MRI is known to be precise and equivalent to the programmed pixel size [51]. In practice, the geometric accuracy is known to be sub-millimeter [52]. The vessel networks in each image are clearly visible due to the sharp image contrast provided by the presence of the contrast agent, which makes the blood appear bright relative to dark non-blood tissues. We averaged each 2 × 2 × 2-voxel cube of adjacent voxels into a single 1.4–1.8 mm voxel to remove noise, reduce processing time, and match conventionally-acquired resolutions. This reduced noise-induced errors without substantially changing the number of vessel segments represented. In two of our 20 image volumes, segmentation failed because bright, non-blood tissues were present very close to the blood volume, so that no threshold value excluded all non-blood objects as described in S1 Text. We did not record any vessel segments from these image volumes. We saw no relationship between failure of segmentation and vascular system geometry.

### Software and Algorithm

**Fig 6:**
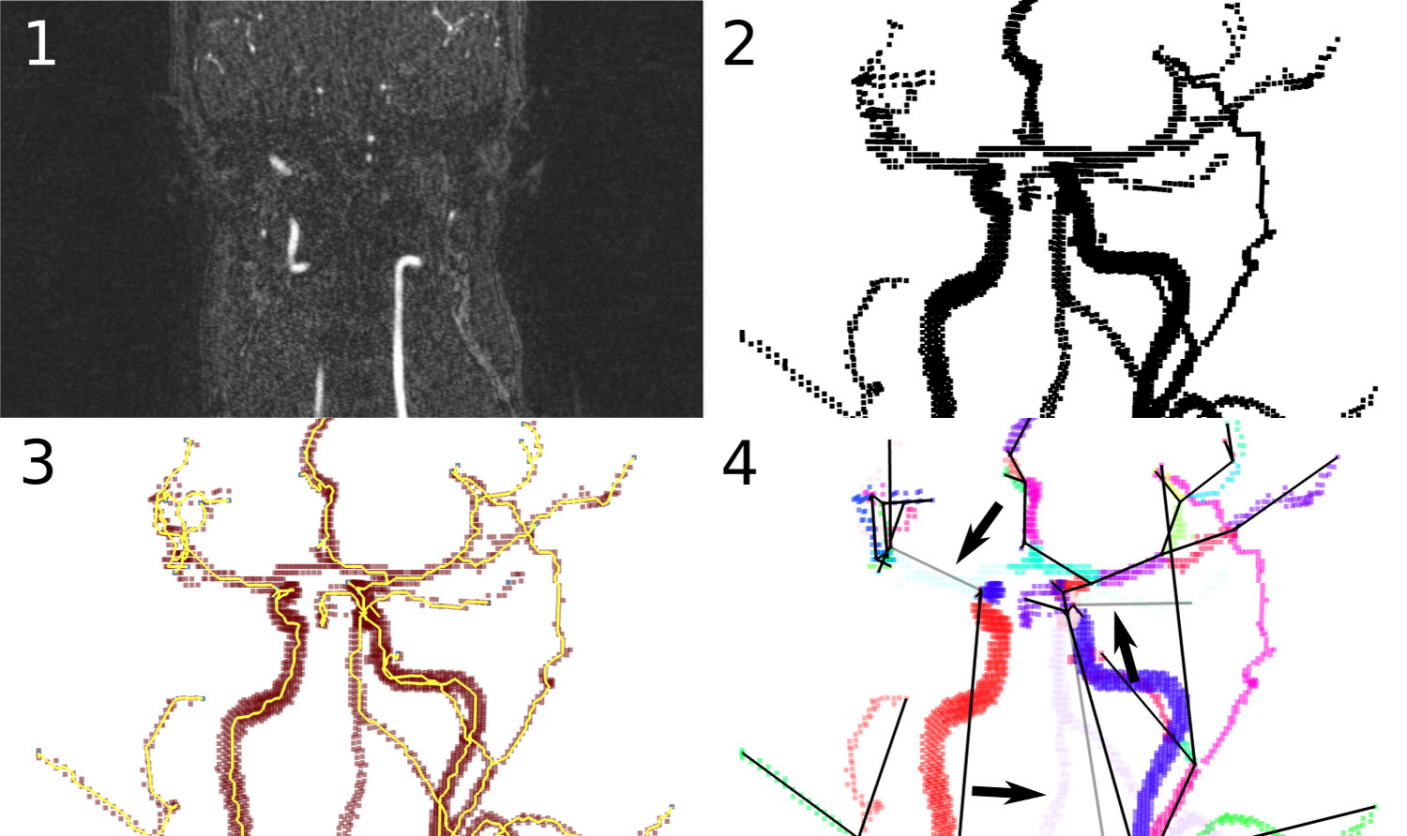
Images from each step of the automatic vessel segmentation process implemented by our software, angicart. 1. Midplane 2D slice from MRI input (original imagery). The input to the software is any tomographic imagery, regardless of imaging method. MRI, CT, and MicroCT are all possible data sources. 2. The network mask (3D rendering of included points). This is the largest group of connected pixels that exceed an intensity threshold. 3. The skeleton. Angicart skeletonizes the vessel network by removing any (red) points that do not disconnect the endpoints (pale blue). The points that remain after this process is completed are the centerline (yellow). Branch points are those with more than two neighboring voxels, and endpoints are those with only one neighboring voxel. 4. Vessel segment decomposition. Angicart partitions the skeleton into vessel segments. Each segment is colored randomly, and black lines are used as a simple map of the endpoints of each segment. Some segments (depicted translucent and indicated by black arrows) are ignored by the analysis, in this case due to reticulation present in normal human anatomy.

We created a free, open source software package—angicart—to read tomographic images of vascular networks, to automatically decompose a vessel lumen into vessel segments, and to measure the geometry and topology of the segments (Fig 6). The software and data used in this study are available on the internet (https://github.com/mnewberry/angicart/) under a GNU Public License. Our software starts by classifying voxels in a 3D image as part of the vessel lumen if they are within the largest connected group of voxels that exceed an intensity threshold [30, 53], as with other level set or thresholding methods. We use a manual binary search to select the threshold value that best matched visual identification of vessels. The result is a 3D binary image, called the *network mask*. Next, we use spatial criteria to find the endpoints of the vessel network, where the vessels become too small to detect. Given these endpoints, we find the centerline and branch points of vessels by skeletonization [54, 55] (See S1 Text). We implement skeletonization using an erosion technique—successively removing voxels until no more are removable without disconnecting the endpoints in the mask [56]. The voxels that remain after erosion lie within approximately 1 voxel-width of the true centerline.

This information allows us to partition the network mask into segments by attributing each voxel to the segment whose centerline is closest to it. We record how vessels are connected and measure the length of each segment’s centerline and the volume of each segment. Following a geometric argument (see S1 Text), quantization error leads us to overestimate length. We compute radius as $r=\sqrt{V/\pi l}$. Radii are therefore underestimated on average due to the quantization error in length measurements. This bias affects the accuracy of individual length and radius measurements, but does not bias estimates of scaling exponents (relative measures) as long as the percent error does not change systematically with vessel size, which it does not. As a final filter of possibly misclassified vessels, we omit vessels in which more than 20% of the voxels lie further than (*r* + 1) from the centerline, or whose total volume is less than 4 voxels. Further details of each step are presented in the S1 Text.

### Data Fitting

We determined the conservation-based node scaling exponents by solving Eq (1a) numerically using Newton’s method implemented in OCaml [57] and iterated until the sum of powers was within 0.00001 of 1. We estimated the regression-based scaling exponents using Standard Major Axis (SMA) regression of the natural log of radius (length) against the natural log of the number of downstream endpoints [58]. We used SMA regression because the variability and uncertainty in the y-axis (vessel radius or length) is as large as the variability and uncertainty in the x-axis (number of downstream endpoints). Furthermore, SMA is appropriate because our goal is to obtain the best estimate of the scaling exponents (slopes) and not the best prediction of *y* given *x*.

We estimated distribution-based scaling exponents by fitting the tail of the probability distribution of radius and length to a power law. That is, we binned log-transformed data and determined the slope of the log of probability density versus the log of radius and length using SMA regression [58]. We used 20 bins and discarded 5 and 7 initial bins of radius and length respectively. Blood vessels near the resolution limit of MRI may not be visible. Although dimensions measured from observed small vessels are used in our other methods, counts of small vessels are unreliable due to censoring. Thus, we discarded initial bins in order to exclude vessel sizes where non-uniform censoring of values might occur. We computed standard errors by varying bin size and the number of initial bins discarded (up to ±3 each) and using the middle 95th percentile of these values. By binning our data and fitting the power law using SMA regression, we avoid problems that can arise when using maximum-likelihood estimators to fit our power-law distributions. Specifically, the maximum-likelihood estimators are derived with specific assumptions and support choices such as smooth and continuous or discrete and integer, as in Clauset et al. [59]. In contrast, our distributions may be somewhere in between: continuous with an increased likelihood to take values near certain points, such as powers of *β* times the aorta radius. Our SMA regression on bins of simulated vessel data produced stable estimates with relatively little bias in comparison to fits based on published maximum-likelihood estimators.

## Supporting Information

S1 Text Detailed Methods and Sensitivity Analysis. (PDF)

S1 Fig Sensitivity of the measured scaling exponents to added image noise ranging from 1 to 10 times the baseline rate (0.47%). The top left panel shows the number of vessel segments used to calculate the four scaling exponents for vessel radius versus the magnitude of the added noise. The top right panel shows the number of vessel segments used to calculate the four scaling exponents for vessel length versus the magnitude of added noise. The bottom left panel depicts how the four calculated scaling exponents for vessel radius vary with the magnitude of added noise. The horizontal lines indicate the WBE predictions for large and small vessels. The bottom right panel depicts how the four calculated scaling exponents for vessel length vary with the magnitude of image noise. The horizontal line indicates the WBE prediction.
(PDF)

S1 Dataset Vessel Segment Dataset. The complete, raw output of angicart on the 18 images used in the study, in tab-separated values (tsv) format.
(TSV)
