# Supplementary material for: Testing Foundations of Biological Scaling Theory Using Automated Measurements of Vascular Networks
Source: PLoS Comput Biol. 2015 Aug 28;11(8):e1004455. doi: 10.1371/journal.pcbi.1004455 (PMC4552567; doi:10.1371/journal.pcbi.1004455)
Supplement: S1 Text — (PDF) [file pcbi.1004455.s001.pdf]

S1. Electronic Supplementary Appendix

S1.1. **Network Mask.** The first step in our process is to obtain a binary mask (or set of voxels) that describes the shape of the blood vessel network. Separating a structure of interest from the background is known in the computer vision literature as segmentation[56]. The literature on image segmentation is voluminous and includes studies specifically examining segmentation of tomographic images of blood vessels. There are many approaches to segmentation of blood vessels, including region-growing, ridge-tracing, watershed models, spectral approaches, and deformable contours[54]. We chose a threshold-based approach for its conceptual simplicity, estimability of errors, and lack of implicit assumptions about blood vessel geometry. There are more sophisticated approaches, but they rely on circularity[S1], volume-servicing[S2], or other assumptions to improve their visual quality. We avoid these assumptions as they might bias the output in favor of models our measurements are designed to test. By using simple thresholds rather than Bayesian classification, we remain agnostic to the image capture method by ignoring the physics of the imaging system.

To select a mask representing the blood vessel network, we calculated the set of voxels that passed an image-specific intensity threshold, then chose the largest connected group of such voxels. To identify the largest connected group, and at many other steps of the analysis, we consider a set of voxels as an adjacency graph. In an adjacency graph, the nodes are the voxels, and the edges are connections between neighboring nodes. We considered two voxels to be connected if they were adjacent in any sense, that is, the voxels touch at at least one corner. The distance along the edge is the distance between the center points of neighboring voxels. In the language of graph theory, the largest connected group of voxels that pass a given threshold is the largest connected component of the adjacency graph. A typical network mask is shown in panel 2 of Fig 6. We use only the largest connected component because smaller groups of voxels are either noise, patient motion artifacts, fatty tissue, or isolated vessels whose relationship to the rest of the network cannot be determined. Finding the largest connected component is the rate-limiting process at this stage, but its time is still $O(N \ln(N))$ for an N-voxel image.

The threshold parameter affects how much of the network is visible. The value affects the volume measurement of each vessel segment to some degree, but the magnitude is expected to be small[53]. At lower (more permissive) thresholds, more of the network is visible, but dimmer objects that are not blood are more likely to be part of the largest connected component. Fortunately, these misidentified objects are readily identifiable to a human observer, allowing us to easily choose a threshold at which no such objects appear. The optimal threshold is the lowest threshold that does not misidentify any objects. We chose an optimal threshold for each image using a manual binary search.

S1.2. **Endpoint Identification.** The skeletonization process removes voxels from the network mask until only those required for maintaining a single connected component remain. Without some initial set of non-removable nodes, this process would remove all nodes. Therefore, it is important to reliably determine a set of non-removable nodes – network endpoints that represent the most distal visible part of each vessel branch. Given the network endpoints, skeletonization will reduce the network mask to centerlines, but skeletonization itself cannot identify the endpoints because they may be removed without disconnecting the graph. Failure to identify endpoints before skeletonization results in the loss of vessel segments from detection (see Methods).

We identify endpoints as the local maxima of a distance transform starting from an interior

voxel. Distance transforms are discussed in the next section. This transform assigns higher values to voxels that are more distal in the network, measured along the contours of vessels. Voxels at a local maximum of distance are the network endpoints. We consider a local maximum to be any voxel whose distance value is greater than all of its neighbors. Since this sometimes leads to multiple endpoints in a single terminal vessel, we collapse endpoints that are within the largest vessel radius (7 mm) of one another and joined by a straight line through the network mask.

S1.3. **Skeletonization.** We use skeletonization to identify vessel centerlines and branch points. A skeleton is an irreducible set of voxels that connect a set of endpoints. That is, the removal of any non-endpoint voxel in the skeleton breaks its adjacency graph into at least two connected components. Thus, we compute our skeleton by recursively removing (eroding) voxels from the network mask, provided that they do not break the new mask into two components. There typically does not exist a unique skeleton for a given network mask and set of endpoints. We chose a particular skeleton in which the remaining voxels conform to the blood vessel centerlines by preferentially removing nodes from the outside (surface) of the mask first.

To remove outermost voxels first, we use a distance transform to rank the voxels according to a measure of how close they are to the outside of the mask. We chose a measure that is lower for voxels closer to the exterior of the mask, and lower when a voxel has more neighboring voxels outside the mask. By removing voxels with more outside neighbors first, we remove voxels at convex points on the surface, smoothing the surface during erosion. A distance transform $D(v)$ of a voxel v has the property that

$$D(v) = \min_{n \in N(v)} \{\mathrm{Dist}(v, n) + D(n)\}, \tag{1}$$

where $N(v)$ is the set of v's 26 neighbors and $\mathrm{Dist}(v, n)$ is the Euclidean distance from v to a neighbor n, which depends on whether the neighbors share one, two, or four corners (i.e., a face). This property is valid except when v is the origin or part of an initial boundary condition. Given a value $D(v)$ at any set of voxels, this property (1) can be used to extrapolate the distance transform to any connected set of voxels using a greedy algorithm. We use an analog of Dijkstra's algorithm[S3]. We supply the values $D(v)$ at points on the surface — the boundary condition. We choose the boundary to be the set $B = \{v \text{ such that } N(v) - M \neq \emptyset\}$ of voxels with any neighbors outside the network mask M, and define $D(v)$ on the boundary to be

$$\left(\sum_{n \in N(v) - M} \frac{1}{\mathrm{Dist}(v, n)} \right)^{-1}.$$

This inverse sum of inverses assigns lower scores to voxels with more neighbors outside the network mask.

Erosion requires checking each voxel for whether its removal disconnects the remaining voxels. Calculating the number of connected components of the remaining voxels is computationally expensive ($O(N \ln N)$), making the erosion process notoriously slow[55]. However, we greatly speed our algorithm by exploiting the fact that removal of a voxel cannot disconnect the whole graph unless it disconnects its neighbors. Checking the last condition is fast ($O(1)$). A few passes of removing such voxels eliminate most of the voxels in the network, creating a close approximation to the skeleton. To erode the last few removable voxels, we use the whole-network algorithm, calculating connected components for each remaining voxel. This novel algorithm produces a skeleton in a small fraction of the time.

Because the skeleton is not unique, different erosion algorithms result in different skeletons.

For our 20 images, our erosion leads to adequate representations of vessel centerlines. We expect any erosion algorithm that preferentially removes voxels roughly according to the above criteria to well-represent the centerlines and give similar results.

S1.4. **Segment decomposition.** Using the skeleton, we can detect which voxels correspond to the branch points of the blood vessel network. The skeleton itself is a set of voxels, and hence its adjacency graph is not a tree because small cycles occur near branch points. Thus, we first compute a minimal spanning tree of the skeleton. In this tree, nodes with more than two neighbors exactly correspond to branch points, and nodes with only one neighbor are vessel endpoints. Branch points and endpoints demarcate vessel segments. We consider the length of a vessel segment to be the distance along the tree between the segment's ends. This distance is the sum of distances between the centers of adjacent voxels, which are either 1, $\sqrt{2}$, or $\sqrt{3}$ times the voxel width. This path length along the voxel grid is greater than or equal to the Euclidean distance between two points, and thus quantization of the vessel centerline will cause lengths to be overestimated in comparison to other studies that do not require vessel contours to conform to the grid[S4][S5]. Much as with the Manhattan metric (discrete l_1 norm), the ratio between the grid distance and Euclidean distance is independent of the scale of the grid. That is, at any given orientation relative to the grid, the bias is a fixed ratio independent of line length. This error is correctable with smoothing, but because our use of length measurements is to determine scaling exponents (relative measures), our results are not affected by biases that increase length of vessels by a constant factor. Consequently, we choose to ignore biases of this type.

Given the centerlines of vessel segments, we can attribute each voxel in the network mask to the vessel segment whose centerline lies closest to it. To accomplish this we use Dijkstra's algorithm to generate a shortest path tree in which any path along the center lines is zero distance. Removing all branch points then breaks the shortest path tree into one connected component per vessel segment. The connected component of each segment contains all information about the segment size, shape, and spatial position. The volume of a segment is the number of voxels multiplied by the volume of each voxel. We compute the radius of each segment from the length and volume as $r = \sqrt{V/\pi l}$. This is effectively equivalent to averaging multiple measurements of radius along the vessel, resulting in a low error in radius. We also label vessel segments and record their topology to compute the number of downstream endpoints, scaling exponents a and b, and scaling ratios β and γ.

Segment decomposition leads to erroneous vessel segments when: 1. a closely-spaced bundle of vessels is identified as a single vessel; 2. endpoint identification misses an endpoint; 3. the network mask contains a loop; or 4. patient motion artifacts cause large volumes of blood to appear as patches that are skeletonized as individual blood vessel segments. Case 1 introduces segments with erroneously large radii into the distributions, but it occurs rarely, and usually in conjunction with a loop. Cases 2 and 3 cause one segment to be missed, and its voxels attributed to adjacent segments, but these malformed segments are detectable. Most of the voxels of tubular vessel segments are within a distance from the centerline that is less than the average vessel radius. We eliminate any vessel in which more than 20% of the voxels are further than $(r + 1)$ from the centerline, or whose total volume is less than 4 voxels. This criterion also eliminates most of the vessels erroneously introduced in case 4.

References

S1. Aylward SR, Bullitt E. Initialization, noise, singularities, and scale in height ridge traversal for tubular object centerline extraction. Medical Imaging, IEEE Transactions on. 2002;21(2):61–75.

S2. Jiang Y, Zhuang Z, Sinusas AJ, Papademetris X. Vascular tree reconstruction by minimizing a physiological functional cost. In: Computer Vision and Pattern Recognition Workshops (CVPRW), 2010 IEEE Computer Society Conference on. IEEE; 2010. p. 178–185.

S3. Sniedovich M. Dijkstra's algorithm revisited: the dynamic programming connexion. Control and Cybernetics. 2006;35(3):599.

S4. Antiga L, Steinman DA. Robust and objective decomposition and mapping of bifurcating vessels. Medical Imaging, IEEE Transactions on. 2004;23(6):704–713.

S5. Kline TL, Zamir M, Ritman EL. Accuracy of microvascular measurements obtained from micro-CT images. Annals of Biomedical Engineering. 2010;p. 1–14.
