# Supplementary figures and images for: Testing Foundations of Biological Scaling Theory Using Automated Measurements of Vascular Networks
Source: PLoS Comput Biol. 2015 Aug 28;11(8):e1004455. doi: 10.1371/journal.pcbi.1004455 (PMC4552567; doi:10.1371/journal.pcbi.1004455)

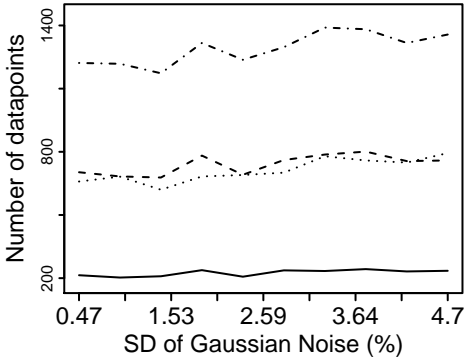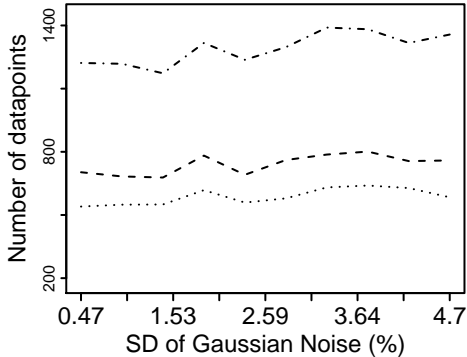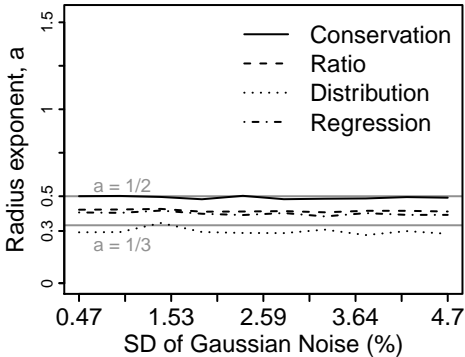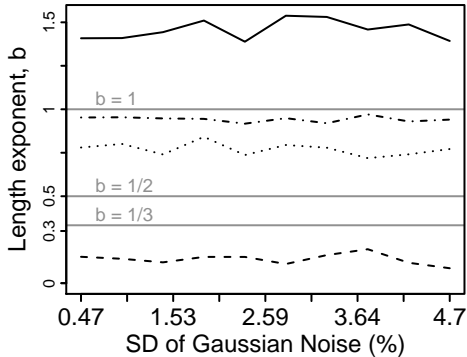

Supplement: S1 Fig — The top left panel shows the number of vessel segments used to calculate the four scaling exponents for vessel radius versus the magnitude of the added noise. The top right panel shows the number of vessel segments used to calculate the four scaling exponents for vessel length versus the magnitude of added noise. The bottom left panel depicts how the four calculated scaling exponents for vessel radius vary with the magnitude of added noise. The horizontal lines indicate the WBE predictions for large and small vessels. The bottom right panel depicts how the four calculated scaling exponents for vessel length vary with the magnitude of image noise. The horizontal line indicates the WBE prediction. (PDF) [file pcbi.1004455.s002.pdf]
